# Environmental and Ecological Risk Assessment of Trace Metal Contamination in Mangrove Ecosystems: A Case from Zhangjiangkou Mangrove National Nature Reserve, China

**DOI:** 10.1155/2016/2167053

**Published:** 2016-10-04

**Authors:** Jun Wang, Huihong Du, Ye Xu, Kai Chen, Junhua Liang, Hongwei Ke, Sha-Yen Cheng, Mengyang Liu, Hengxiang Deng, Tong He, Wenqing Wang, Minggang Cai

**Affiliations:** ^1^Coastal and Ocean Management Institute, Xiamen University, Xiamen 361102, China; ^2^Fujian Provincial Key Laboratory for Coastal Ecology and Environmental Studies, Xiamen University, Xiamen 361102, China; ^3^College of Ocean and Earth Science, Xiamen University, Xiamen 361102, China; ^4^Xiamen Environmental Protection Bureau, Xiamen 361004, China; ^5^College of Ocean Science and Resources, National Taiwan Ocean University, Keelung 202, Taiwan; ^6^College of Environment and Ecology, Xiamen University, Xiamen 361102, China; ^7^State Key Laboratory of Marine Environmental Science, Xiamen University, Xiamen 361102, China

## Abstract

Zhangjiangkou Mangrove National Nature Reserve is a subtropical wetland ecosystem in southeast coast of China, which is of dense population and rapid development. The concentrations, sources, and pollution assessment of trace metals (Cu, Cd, Pb, Cr, Zn, As, and Hg) in surface sediment from 29 sites and the biota specimen were investigated for better ecological risk assessment and environmental management. The ranges of trace metals in mg/kg sediment were as follows: Cu (10.79–26.66), Cd (0.03–0.19), Pb (36.71–59.86), Cr (9.67–134.51), Zn (119.69–157.84), As (15.65–31.60), and Hg (0.00–0.08). The sequences of the bioaccumulation of studied metals are Zn > Cu > As > Cr > Pb > Cd > Hg with few exceptions. Cluster analysis and principal component analysis revealed that the trace metals in the studied area mainly derived from anthropogenic activities, such as industrial effluents, agricultural waste, and domestic sewage. Pollution load index and geoaccumulation index were calculated for trace metals in surface sediments, which indicated unpolluted status in general except Pb, Cr, and As.

## 1. Introduction

Differing from the land ecosystem and marine ecosystem in the structure and function, the mangrove wetland, periodically suffering seawater immersion, locates in the dynamic ocean and land interface of tropics and subtropics and plays an extremely important role in the global ecological balance [1, 2]. In recent years, since many mangrove ecosystems are close to urban development which is tied to industrialization and human activities, the important intertidal zone is subjected to contamination from a variety of human activities [3–5], because it is always located near the coast where the wind is weak and the water movement is slow and the pollutants can directly settle in the sediments [6]. Inherent physical and chemical properties of mangrove sediments confer an extraordinary capacity to accumulate materials or pollutants discharged to the nearshore marine environment [7, 8].

Because of the toxicity, broad sources, biodegradable properties, and cumulative behavior, trace metals are of special environmental concern [9]. Trace metals with low solubility in water are easily adsorbed and accumulated in sediments [10]. Therefore, coastal sediments are always regarded as the ultimate sinks for trace metals [9]. On the other hand, some trace metal elements cannot be permanently fastened by sediments and will be released back to the overlying water, when the environmental condition changes (like salinity, resuspension, pH, redox potential, and the organic matter decay rate) [11, 12].

Some trace metals such as copper, zinc, cobalt, and arsenic are essential elements of marine organisms in the environment, indispensable for body for normal physiological activity [13]. Nevertheless, trace metals are such as cadmium and mercury, which have no essential biological functions and are toxic even at low concentrations [14, 15]. The organism is exposed to these pollutants from the water or the particles and then accumulates them in the body [16–18]. Humans, as a final link in the food chain, are always mostly affected, and consequently the public has become the potential risk to human health when consuming such polluted biota [19]. Conversely, benthic organisms (like gastropod, fish, and crab) can be used to monitor the quality of aquatic ecosystems with broad geographical distribution, fixed lifestyle, easiness of capture, and the promotion of bioturbation [17, 18, 20, 21].

Although several studies have focused on trace metal distribution in mangrove sediments and benthic organism worldwide [22–26], little is known about mangroves in the southeast China [27–29], where high levels of trace metals were observed in the biota according to the rather limited studies [30–32]. From 1980 to 2006, some researches have been conducted to investigate the content and distribution of trace metals in the surface sediments in Dongshan Bay and Zhangjiang River Estuary mangrove zone to examine the concentrations of some selected trace metals (Cr, Cu, Zn, As, Cd, Hg, and Pb) in the surface sediment [6, 33–35]. However, no reports are available in the Zhangjiangkou Mangrove National Nature Reserve (ZMNNR) relating metals in the sediments to bioaccumulation in edible biota [27–29]. With more industry developed nearby, such as nuclear power industry, chemical industry, and power plants, it is necessary to investigate and assess the environmental risk in Zhangjiang River catchment for environment management and sustainable development [5, 36].

The objectives of this study were to measure the contents and distribution of some trace metals in surface sediments and benthic animals from ZMNNR. The goals of this research were (1) to evaluate the concentration level and distribution of the trace metals in the sediment and biota in the ZMNNR; (2) to accurately quantify the extent of trace metal pollution using the geoaccumulation index (*I*_geo_) and pollution load index (PLI); and (3) to analyze possible sources of trace metals in sediment using the Pearson correlation analysis, cluster analysis (CA), and principal component analysis (PCA).

## 2. Materials and Methods

### 2.1. Study Area

Zhangjiangkou Mangrove National Nature Reserve, covering approximately an area of 23.60 km^2^, is the biggest mangrove zone to the north of the Tropic of Cancer, China. ZMNNR locates at the outlet of the Zhangjiang River (23°23′–23°56′ N, 117°24′–117°30′ E), which flows into the Dongshan Bay from the northwest, and is a traditional agriculture area, which irrigates vast stretches of farmland along its course. The catchment area and total length are 855 km^2^ and 58 km, respectively. Dongshan Bay locates on the west coast of the south of the Taiwan Strait, covering 247.89 km^2^ area, with a water surface of approximately 155.5 km^2^, and is the largest bay in southern Fujian. The bay is a semiclosed bay, inundated by the incoming tide twice a day, with the largest tidal difference during spring at 3.15 m. The current of the bay is a reciprocating semidiurnal tidal current. The bay is a place in which the East China Sea and South China Sea cross where the fish from East China Sea and South China Sea fish can be bred.

The average temperature is between 13.5°C (in January) and 28.9°C (in August) in this area. The wind direction during the dry season is prevalent from north-west to south-east while it is the opposite during the rainy season. Annual fall of rain is about 1714.5 mm and the monsoon will bring heavy rainfall during rainy season from April to September.

### 2.2. Sampling

A lot of research elaborates the method for extraction, purification, and measurement of trace metals in sediment and biota samples elsewhere, including Cai et al. [6] and Shi et al. [43], to name but a few. And we give only a concise presentation here.

**Figure 1:**
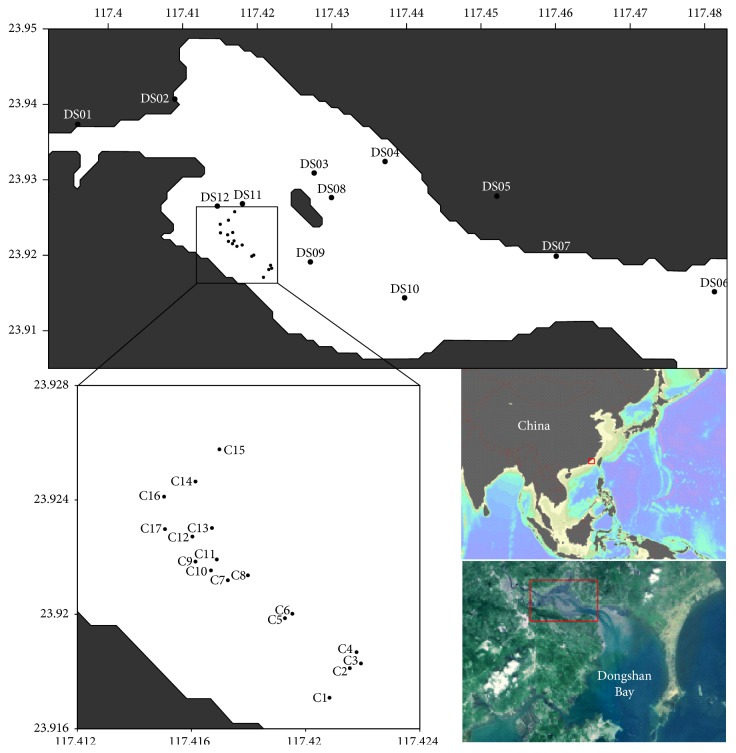
The study area and sampling stations.

29 surface sediment samples and the biota specimen were carried out from the study area in August 2013 (Figure 1). In this study, the sampling region included the mangrove intertidal zone (C01–C17) and the subtidal zone (DS01–DS12). Surface sediments were collected with a grab sampler and were subsampled from the center of the collected material using plastic spatula. Once removed the samples were placed into precleaned Teflon containers and stored at −20°C before undergoing analysis in a laboratory.

The benthic, mussel (*Siliqua minima*,* SM*), crab (*Uca maracoani*,* UM*), and fish (*Boleophthalmus pectinirostris, BP*) of biota specimens were also carried out from the study area in August 2013. The two kinds of biological samples were sampled by local fisherman from northwest Zhangjiang River. The fishes were bought from the local fisherman on the same day of capture and brought to the laboratory, where they were stored at −20°C until analysis. During analysis stage, we selected the samples (*Siliqua minima*, *n* = 25;* Uca maracoani*, *n* = 10) in the medium body length and preferable growth situation. The samples had been dissected to separate their shell and tissue, freeze-dried, and stored at −20°C.

### 2.3. Sample Analysis

 The sediment samples for trace metals analysis were freeze-dried under −80°C for 36 hours and then ground to powder using an agate mortar and pestle and passed through a 180-mesh nylon sieve to remove large particles. The screened sample was collected in acid-rinsed glass vials and stored in desiccators. 0.1000 ± 0.005 g of sediment sample (dry weight) was put into acid-washed PTFE vessel and digested with the method of HNO_3_ + HClO_4_. Sample was diluted to 50 mL with Milli-Q water (National Standard of China, GB 17378.5-1998). Sample solutions were analyzed for Zn using flame atomic absorption spectrometry (FAAS, SOLAAR M6, Thermo Electron) and for Cr, Pb, Cu, and Cd using Graphite Furnace Atomic Absorption Spectrometer (GF-AAS, SOLLAAR M6, Thermo Electron) and for Hg and As using inductively coupled plasma mass spectrometry (ICP-MS) (DRC-II, Perkin Elmer, USA). 0.200 ± 0.005 g of freeze-drying of benthic animals sample was put into acid-washed PTFE vessel and digested with the method of HNO_3_ + H_2_O_2_ (National Standard of China, GB 17378.6-2007). Sample solutions were analyzed for Cu, Cr, and Cd, using Graphite Furnace Atomic Absorption Spectrophotometer (GF-AAS).

Granulometry was analyzed using a laser particle size analyzer (Mastersizer 2000, Malvern) and pH was performed by Vario pH meter (SMCH-2V00-001V, German).

### 2.4. Quality Assurance and Quality Control (QA/QC)

All reagents were of superior grade pure and ultrapure water throughout this study using a Milli-Q water purification system (Millipore, Bedford, MA, USA) with resistivity of 18.2 Megohm-cm. A method blank, limit of detection (LOD), and precision were run to correct the measurement. Lab ware prepared for sample was soaked in nitric acid (1 : 3) for at least 48 h and then rinsed with Milli-Q water. Detection limits were 1.80 *μ*g/L for Zn, 0.03 *μ*g/L for Cr, 0.21 *μ*g/L for Pb, 0.009 *μ*g/L for Cd, 0.06 *μ*g/L for Cu, 0.04 ng/g for As, and 0.05 ng/g for Hg. All samples were analyzed in duplicate. Sediment standard reference material (GBW07314; National Research Council of China) was digested in quadruplicate and analyzed to support the QA/QC of sediment measurements, which yielded satisfactory results, with recovery ranging from 85% to 115%. Biota standard reference material (DORM-4, National Research Council of Canada; GBW10024, National Research Council of China) was digested in quadruplicate and analyzed to support the QA/QC of biota measurements, which yielded satisfactory results, with recovery ranging from 81% to 112%.

### 2.5. Quantification of Sediment Pollution

#### 2.5.1. Pollution Load Index (PLI)

 To assess the level of trace metal pollution, an integrated pollution load index of eight metals was calculated as suggested by Suresh et al. [44]. PLI > 1 means that pollution is present; otherwise, if it is below 1, there is no metal pollution

(1) $$\begin{array}{c} \text{PLI}={\left(\;{\text{CF}}_{1}*{\text{CF}}_{2}*{\text{CF}}_{3}*\cdots *{\text{CF}}_{n}\;\right)}^{1/n}. \end{array}$$

The contaminant factor (CF) was developed by Hakanson [45] and had been widely used in trace metals studies of sediments and soils [46].

#### 2.5.2. Geoaccumulation Index (*I*_geo_)

The geoaccumulation index (*I*_geo_) was introduced by Müller [47] to quantify trace metal pollution levels in the study area. The index can be calculated by the following expression:

(2) $$\begin{array}{c} I_{\text{geo}}=\log_{2}\left(\;\frac{C_{n}}{1.5B_{n}}\;\right), \end{array}$$

where *C*_*n*_ is the measured concentration of trace metal in the mangrove sediment and *B*_*n*_ is the geochemical background value in average shale. Because there is no background value of metal in the sediment from the study area, the geoaccumulation index was calculated using the values of the earth's crust [48].

#### 2.5.3. Biota-Sediment Accumulation Factor

To estimate the proportion in which metal occurs in the organism and in associated sediment, biota-sediment accumulation factors (BSAFs) were calculated for selected metal in the molluscs studied by the following expression:

(3) $$\begin{array}{c} \text{BSAF}=\frac{C_{x}}{C_{s}}, \end{array}$$

where *C*_*x*_ is the mean concentrations of metals in the organism, *C*_*s*_ is the mean concentrations of metals in associated sediment [49, 50].

### 2.6. Statistical Analysis

Pearson correlation analysis, cluster analysis, and principal component analysis were conducted using SPSS® for Windows Release 21.0 (SPSS Inc., US). Pearson correlation analysis and cluster analysis were applied to examine the relationship among trace metals in the surface sediment. The general characteristics of the ZMNNR sediments were further analyzed using PCA methods to determine the degree of pollution by trace metals from natural (lithogenic) action and anthropogenic sources [51–53].

## 3. Results

### 3.1. Trace Metals in the Surface Sediments

Major sediment characteristics are shown in Table 1, which presents the granulometry, the content of water (CW), and pH in surface sediments from ZMNNR. The result indicated that the sediment mainly consists of silt and clay-type soil, which account for an average 29.77% and 58.18%, respectively. Sand contents were low (average is 11.84%) except in DS03 and DS07 (74.78% and 45.8%, resp.). Water content in sediments varied from 33.05% to 52.2%, with a mean of 42.92%, and pH ranged from 5.99 to 8.26, with a mean of 7.53.

The concentrations of seven common trace metals (Cu, Cd, Pb, Cr, Zn, As, and Hg) in the sediments from ZMNNR are listed in Table 1. The ranges of Cu, Cd, Pb, Cr, Zn, As, and Hg were 10.79–26.66 mg/kg (21.20 mg/kg in avg.), 0.03–0.19 mg/kg (0.07 mg/kg in avg.), 36.71–59.86 mg/kg (44.02 mg/kg in avg.), 9.67–134.51 mg/kg (30.43 mg/kg in avg.), 119.69–157.84 mg/kg (137.63 mg/kg in avg.), 15.65–31.60 mg/kg (25.07 mg/kg in avg.), and 0.00–0.08 mg/kg (0.02 mg/kg in avg.). The mean concentrations of all trace metals in the surface sediments of the ZMNNR were much higher than their respective background levels (Table 2), and in particular those of Cu and Zn were four times higher than the background levels. This exceeding of trace metals in the sediments from Zhangjiang River Estuary was also reported by Xie et al. [33].

**Figure 2:**
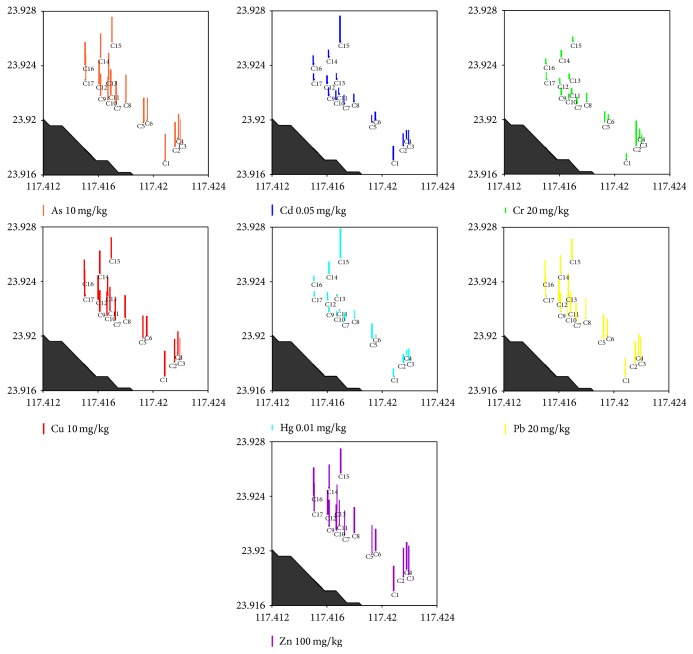
Spatial distributions of trace metals (mg/kg) in the mangrove intertidal zone of the ZMNNR.

**Figure 3:**
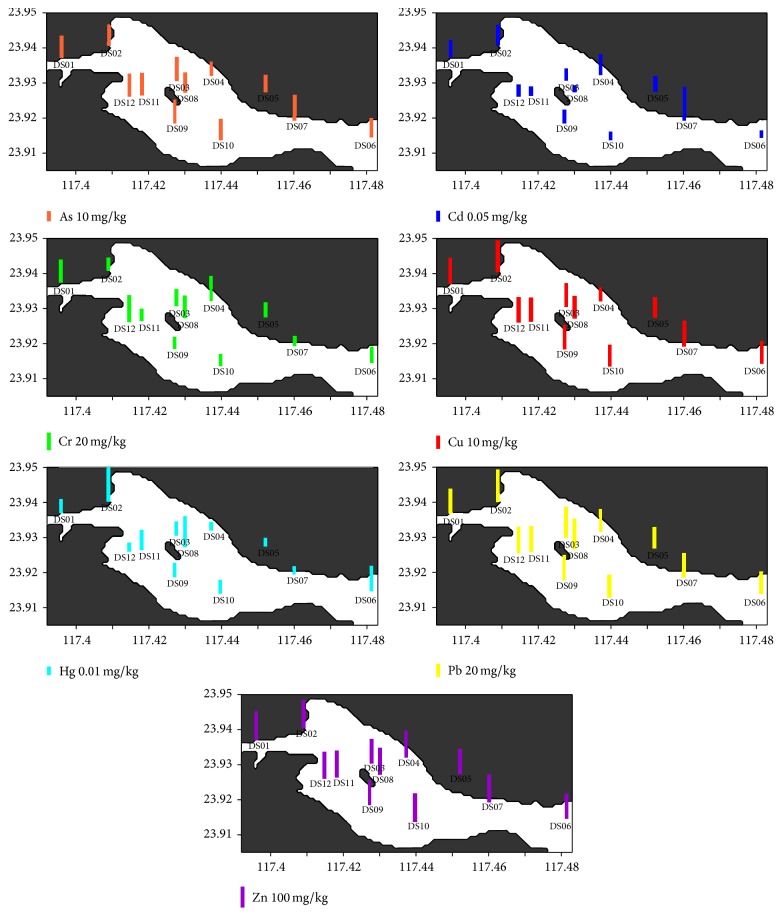
Spatial distributions of trace metals (mg/kg) in the subtidal zone of the ZMNNR.

Furthermore, spatial distributions of trace metals are shown in Figures 2 and 3. Higher concentrations of trace metals are generally found in the fine-grained sediments in the western coastal region of Zhangjiang River Estuary.

### 3.2. Trace Metals Concentrations in Biota

The concentration ranges of different metals in the organism were various. The overall trend of the accumulated degree of trace metals in the samples was as follows: Zn > Cu > As > Cr > Pb > Cd > Hg. Concentrations of four trace metals in the organisms from the study area (Table 3) varied within 1.69~155.99 mg/kg for Cu, 17.88–105.34 mg/kg for Zn, 0.45–1.59 mg/kg for Pb, 0.23–12.35 mg/kg for Cr, 2.20–53.98 mg/kg for As, and 0.01–0.02 mg/kg for Hg. For Cu and Cd, the content of the* UM* sample is far higher than other creatures. For Pb, the concentration of the shell sample is more than the muscle sample. For Cr, the concentration in the* BP* shows the highest value of 12.35 mg/kg in* BP* liver.

## 4. Discussion

### 4.1. Influencing Factors on Trace Metals in Sediment

Grain size, CW, and pH are the most important factors which control the distribution of trace metals in sediments. Significant spatial variation was observed, with increased clay content in sediments of the mangrove intertidal zone and the Zhangjiang River areas. Clay mineral and organic matter are the active components in the adsorption process, which is an important way for trace metals to enter into the sediment. Most of trace metals (Cu, Cd, Zn, and Hg) showed an obviously positive correlation with clay and silt contents with the correlation coefficients of 0.22–0.46 and 0.20–0.36 and negative correlation with sand content with the correlation coefficients from 0.08 for Hg to 0.45 for Zn. They indicated that Cu, Zn, Cd, and Hg tended to accumulate in fine particles which might be a major carrier for transporting these metals from rivers to Zhangjiang River Estuary. On the other hand, as for pH, they were higher in the mudflat > forest, but no significant difference was found among different stations. DS06 in Zhangjiang River had the highest pH, while station C16 in the mangrove intertidal zone had the lowest pH. Low pH values are frequently observed in mangrove forest sediments compared to the vegetated mudflat sediments [54]. This is attributed to the microbial decomposition of mangrove litter and hydrolysis of tannin in mangrove plants which releases organic acids [55]. In addition, anthropogenic turbulence would influence the distribution pattern, especially in the southwestern and northwestern coastal region upstream of mangrove, which seems to be the contribution of metals from Zhangjiang River and wastewater discharge of Yunxiao County. Since the mangrove is located near the Zhangjiang River Estuary, trace metals are highly concentrated due to the convergence of several sewage-polluted tributaries.

When compared with historical records in the document published, the mean concentration of Cu, Cd, Pb, Cr, Hg, and Zn in the ZMNNR surface sediments decreased over the last decade, but the mean concentration of As increased (Table 2). High As concentration might be attributed to the anthropogenic activities such as treatment of agricultural land with fertilizers and arsenical pesticides [56]. Attention should be paid when comparing the historical data on the concentrations of metal pollutants in sediments, because the differences in the pretreatment and analytical methods could cause errors. The same sampling and analytical methods were used in 2007 and 2013 surveys (Table 2). The results showed a decrease in the concentration of Pb, Cr, Cd, and Hg in the ZMNNR sediments. The concentrations of As, Cu, and Zn in the ZMNNR surface sediments decreased from 2007 to 2013.

### 4.2. Assessment of Sediment Quality

Compared with the Interim Sediment Quality Guidelines (ISQG), the average concentrations of these trace metals were over the ISQG-low value, but below the ISQG-high value [37]. The extent of metal pollution was accurately quantified using the pollution load index (PLI) and geoaccumulation index (*I*_geo_). The pollution load index values of Cr, Cd, Zn, Cu, Pb, As, and Hg in all sediment samples are summarized in Table 4. The pollution load index values of all sites range from 0.38 to 1.10. According to the mean PLI value (0.77), the sediments of Zhangjiang River Estuary are unpolluted. But in C15 and DS02 the PLI value is above 1, indicating there may be some transportation and industrial and human activities in these locations.

The *I*_geo_ values were listed in Table 5. Among the studied metals, the *I*_geo_ values showed the decreasing order As > Pb > Zn > Cu > Cr > Cd > Hg. Owing to the Müller scale, the results of *I*_geo_ values indicated that Cd, Cu, Zn, and Hg showed an unpolluted situation at all stations (*I*_geo_ < 0). Cr showed less extent of pollution at stations C02 (*I*_geo_, 0.29), and other stations showed an unpolluted situation (*I*_geo_ < 0). For Pb, stations C07 and DS02 showed values falling into the uncontaminated to moderately contaminated classes (*I*_geo_, 0.61–1.32). For As, uncontaminated to moderately contaminated classes were showed at stations DS04 and DS07 (*I*_geo_, 0.4–1.42). The results suggested that station C02 was polluted by more trace metals than other stations.

The ecosystem in this study, which covered a wide range of perturbations, from industrial activities to agriculture, fisheries, and transport, held higher Pb, Zn, and As content than other mangrove sediments (Table 6). It is noted that the concentration of Cu and Zn in Australia is very high in the mangroves and *I*_geo_ values must also be high. In contrast, the mangroves in Singapore and France are much less contaminated with trace metals, probably because of better management of anthropogenic sources. Within Asia, the sediment in Zhangjiang mangrove zone is more contaminated with Pb and As than the other cities in China like Pearl River, Hong Kong, and Zhanjiang, likely owing to the rapid socioeconomic development in the region of the ZMNNR.

### 4.3. Sources of Trace Metals in Mangrove Sediment

In order to obtain the possible metal sources of ZMNNR sediment, multivariate statistical analyses were carried out. According to the values of the Pearson correlation coefficients (Table 7), many trace metals are significantly correlated (*p* < 0.05). In this study, the Pearson matrix shows the significantly positive correlation of Pb with Cu (*r* = 0.620) and indicates that these elements have the same source, most likely related to the anthropogenic activities in such a densely populated and agricultural area. Anthropogenic activities such as agricultures are the possible causes; for example, manufacturing fertilizers bring about metals and improve the accumulation and capacity of holding them [57]. Significant correlations between Cr and Zn (*r* = 0.565) indicate that these elements are also derived from the same source.

**Figure 4:**
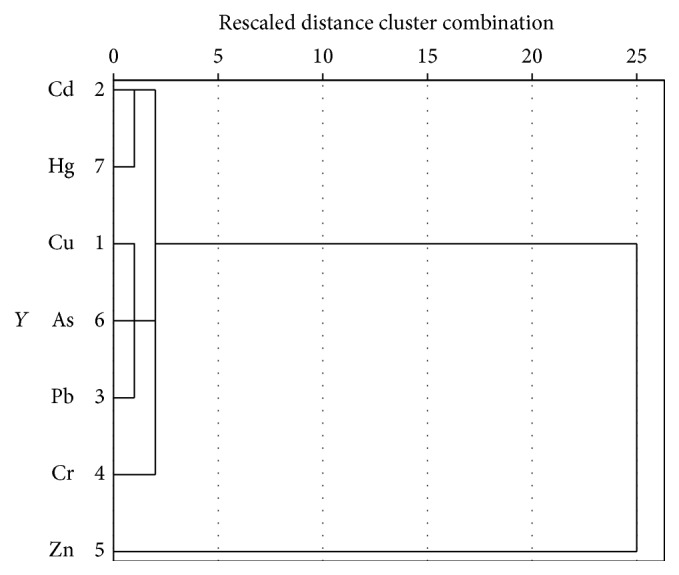
Dendrogram of trace metals concentrations of sediment samples.

Cluster analysis (CA) is a method that is used to provide important information about the grouping of variables on the basis of similarity [58]. It was performed on the data using the Ward method and squared Euclidean distance and grouped into two clusters. It produced a dendrogram as shown in Figure 4. Cluster 1 includes Cd and Hg, which are supposed as contaminants derived from industrial sources. Cluster 2 contains Cu, As, Cr, and Pb which are identified as intensive agricultural activities.

To further explore the extent of metal pollution and for source identification, principal component analysis (PCA) was performed for the studied sediments. And varimax rotation method was used to maximize the sum of the variances of the factor coefficients which better explained the possible sources. The results of PCA for trace metals contents are listed in Table 8. According to these results, Hg, Cd, Pb, Cr, Cu, Zn, and As concentrations could be grouped into a three-component model, which accounted for 72.61% of all of the data variation.

In detail, the principal component 1 (PC1) which has high loading of Cu (0.84), Pb (0.78), and As (0.78) accounts for 28.27% of variance (Table 8) and is the most important component. PC1 could be better explained as the agriculture factor. For irrigation purpose, the farmers often use the mine drainage water and chemical fertilizer that often release some ions in the soil [59]. PC2, which has high loading of Cr (0.87) and Zn (0.86) accounts for 24.23% of the variance. It was in accordance with the results of the correlation analysis (Table 8). These results implied that these metals could be originated from natural source. Xie et al. [33] found that the concentrations of Zn and Cr in ZMNNR sediment were not in evidence with the surrounding human activities. Zn and Cr mainly derived from parent rocks and their distribution patterns may depend on local hydrodynamic conditions [60]. The loading of Cd (0.85) and Hg (0.75) accounts for 20.11% of the variance, where PC3 can be considered as industry component. Pigments, electroplating, and metal industries were probably the major sources of these elements [61].

### 4.4. Comparison of Trace Metals Levels in Tissues

**Figure 5:**
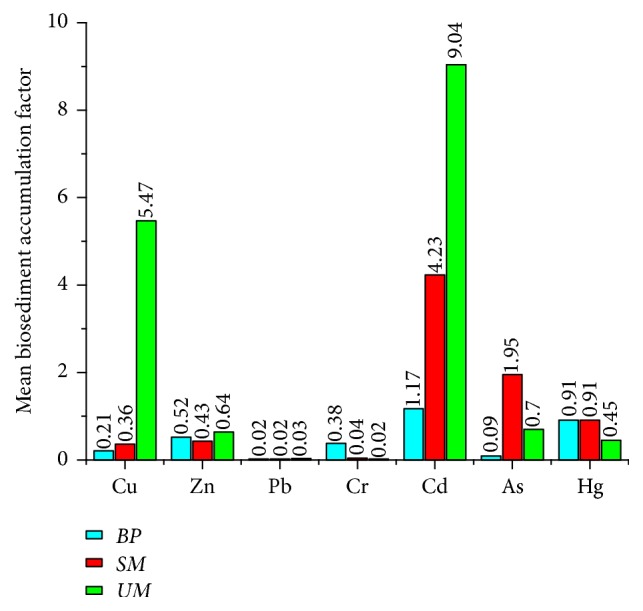
Mean biosediment accumulation factor values (BSAF) in* Boleophthalmus pectinirostris *(*BP*)*, Siliqua minima *(*SM*), and* Uca maracoani *(*UM*).

In order to evaluate the efficiency of trace metals bioaccumulation by* Siliqua minima *(*SM*), the biota-sediment factor, defined as the ratio between metals degrees in molluscs and sediment, respectively [49], has been computed for both species (Figure 5). Cd is the metal which records the highest BSAF values and Pb is the metal with the lowest one.* Uca maracoani* (*UM*) has the highest BSAF mean for all metals except Cr, As, and Hg (Figure 5).

Relative differences in the concentrations of the metals between muscle and liver are apparent, where metal concentrations were between 1 and 5 times higher in* Boleophthalmus pectinirostris*. The reason may be their physiological roles in fish metabolism. As shown in Table 3, the target tissues of trace metals are metabolically active ones, like the liver, kidney, and gill. Therefore, in contrast to some other tissues like the muscle, where metabolic activity is relatively low, metal accumulation in these tissues records higher level [62–64].* SM*, a kind of mussel, is considered as excellent indicators of metal bioavailability in the environment, as the concentrations in its tissues were related to the Bakun upwelling index [65].

Concentrations of Zn and Cu in the tissue of the benthos were higher than those of other metals. The reason may be that Zn and Cu are essential metals for aquatic organisms and thus subject to active absorption in marine organisms [66]. Variations in the concentration of Cu showed a highest level of accumulation in* UM*, whereas concentrations of other metals are appreciably low. Cu is essential for crustaceans, because it can participate in the respiratory pigment hemocyanin [67]. The concentrations of As are of great concern with regard to its richness in the organisms. Cr is an essential trace element in organisms and plays a very important role (in its biologically usable form) during the metabolic processing of glucose [68]. Compared with Zn and Cu, the quantity of Cr demanded for the organisms is low, and thus the levels of Cr in the organisms are relatively lower than those of Zn and Cu. Cd, Hg, and Pb are not essential for organisms and have toxic effects at low concentration [69, 70].

Zn and Cd are the elements that have previously attracted much attention in the ZMNNR, because the concentration often can be related to the intense upwelling of sea water characteristic of the area [20, 71]. The cadmium represents concentrations constantly higher (around 0.29 mg/kg, dry weight) than expected or observed at comparable locations. The highest concentration of Zn was measured in muscle of* SM*. It is worth studying the source of the Cd and Zn enrichment in fish and mussels, respectively. Although industrial pollution, like lead-acid battery factory and chemical industry effluents, can increase the level of contaminant, the possibility of the strong upwelling bringing high levels of Cd and Zn cannot be excluded. Indeed, such a phenomenon has been explicated in the previous study [72].

## 5. Conclusions

We collected the sediment and biota samples in Zhangjiangkou Mangrove National Nature Reserve and conducted the experiment in Marine Organic Chemistry Lab in Xiamen University, Xiamen. The ranges of measured concentrations expressed in mg/kg were the following: 10.79–26.66 mg/kg for Cu, 0.03–0.19 mg/kg for Cd, 36.71–59.86 mg/kg for Pb, 9.67–134.51 mg/kg for Cr, 119.69–157.84 mg/kg for Zn, 15.65–31.6 mg/kg for As, and 0.00–0.08 mg/kg for Hg. Higher concentrations of trace metals are generally found in the fine-grained sediments in the western coastal region of Zhangjiang River Estuary. The results showed a decrease in the concentration of Pb, Cr, Cd, and Hg and increase in the concentration of As, Cu, and Zn from 2007 to 2013 in the ZMNNR sediments. For biota, the overall trend of the degree of accumulation of trace metals in the samples was as follows: Zn > Cu > As > Cr > Pb > Cd > Hg. Moreover, according to data from this study,* Uca maracoani *(*UM*) seems to have a greater bioaccumulation capacity than other species for all the metals (Cu, Pb, Cd, and Zn) considered except Cr. Correlation analysis and PCA illustrated that trace metals (Hg, Cd, Pb, Cr, Cu, Zn, As, and Hg) may derive from metal processing, electroplating industries, industrial and agricultural wastewater, and domestic sewage. And compared to other mangroves all over the world, Pb, Zn, and As are highly enriched in this ecosystem. According to the mean PLI value (0.77), the sediments of Zhangjiang River Estuary are unpolluted except C15 and DS02 station. Owing to the Müller scale, the results of *I*_geo_ values indicated that Cd, Cu, Zn, and Hg showed an unpolluted situation at all stations and that Pb, Cr, and As show polluted situation in some station.

## Figures and Tables

**Table 1:** Physicochemical parameters and trace metals concentrations (mg/kg) in surface sediments from ZMNNR.

Stat.	Clay (%)	Silt (%)	Sand (%)	pH	CW (%)	Trace metals (mg/kg)						
						Cu	Cd	Pb	Cr	Zn	As	Hg
C01	—	—	—	7.93	—	24.96	0.09	36.98	27.53	135.18	26.32	0.02
C02	31.48	63.30	5.22	7.86	44.28	22.78	0.08	42.32	134.51	157.84	24.03	0.02
C03	32.52	63.13	4.35	7.77	40.86	21.44	0.09	47.93	42.30	155.98	24.43	0.03
C04	31.14	66.18	2.68	6.98	40.17	23.13	0.05	42.49	45.03	145.79	24.89	0.01
C05	30.63	66.57	2.80	7.48	39.53	22.85	0.04	50.22	52.55	149.81	25.25	0.04
C06	28.54	67.57	3.89	7.69	42.79	19.68	0.05	36.80	22.07	120.55	22.99	0.00
C07	32.17	64.30	3.53	7.02	41.33	21.86	0.06	36.71	32.39	134.18	23.74	0.02
C08	31.92	65.27	2.81	7.97	39.21	22.43	0.05	41.32	39.13	139.20	28.17	0.02
C09	30.71	65.22	4.07	7.12	44.76	21.30	0.05	39.49	34.25	144.36	22.16	0.01
C10	31.66	63.02	5.32	7.08	41.07	24.54	0.06	48.11	27.73	141.06	23.24	0.01
C11	31.94	63.98	4.08	6.95	44.96	23.84	0.04	39.66	26.72	136.33	25.47	0.00
C12	32.04	63.23	4.73	7.23	41.03	24.68	0.05	41.96	23.12	129.73	22.91	0.02
C13	33.67	62.90	3.43	7.68	49.53	17.14	0.04	46.61	22.93	140.63	27.17	0.00
C14	31.67	65.00	3.33	7.72	39.94	22.57	0.05	38.79	30.98	127.58	24.53	0.03
C15	36.33	63.49	0.18	7.23	40.75	21.02	0.18	41.25	25.60	134.03	25.99	0.08
C16	31.48	63.30	5.22	5.99	49.40	21.02	0.06	42.32	26.59	150.81	23.01	0.01
C17	32.52	63.13	4.35	6.97	—	26.73	0.04	40.48	34.44	150.24	26.16	0.01
DS01	32.75	66.87	0.38	7.45	52.20	21.72	0.09	46.27	27.81	146.22	26.88	0.02
DS02	35.29	59.31	5.40	7.31	41.77	26.66	0.11	59.86	14.42	141.49	26.15	0.07
DS03	8.98	16.24	74.78	7.88	33.05	19.68	0.06	55.31	19.63	119.69	29.27	0.02
DS04	28.31	61.37	10.32	8.22	46.84	10.79	0.11	40.84	29.83	136.76	15.65	0.01
DS05	26.32	49.58	24.10	8.11	44.61	16.64	0.08	36.86	16.61	124.14	19.76	0.01
DS06	21.57	50.03	28.40	8.26	40.12	18.19	0.03	40.84	18.52	124.71	22.51	0.04
DS07	18.79	15.41	45.80	7.97	36.39	21.37	0.19	43.97	9.67	139.77	31.60	0.01
DS08	32.63	59.16	8.21	7.81	44.96	18.48	0.03	56.17	26.60	133.46	24.39	0.05
DS09	26.02	46.93	26.95	7.80	44.46	19.96	0.07	45.38	13.00	122.27	28.72	0.02
DS10	28.18	50.07	21.75	8.11	38.98	18.05	0.04	40.66	13.01	141.20	25.78	0.02
DS11	32.08	61.98	5.94	—	48.61	19.96	0.04	48.51	12.88	133.89	27.83	0.03
DS12	32.20	62.53	5.27	7.12	47.38	21.44	0.06	48.47	32.66	134.32	28.09	0.01

Mean	29.77	58.18	11.33	7.53	42.93	21.20	0.07	44.02	30.43	137.63	25.07	0.02

“—”: no detected.

**Table 2:** Summary of trace metals concentrations in surface sediments of the ZMNNR.

	Time of sampling	Number of samplings	Concentrations (mg/kg)						
			Cu	Cd	Pb	Cr	Zn	As	Hg
This study	2013	17^a^	21.47 ± 2.23	0.064 ± 0.034	41.97 ± 4.09	38.11 ± 26.26	140.78 ± 10.25	24.73 ± 1.67	0.019 ± 0.019
		12^b^	19.41 ± 3.73	0.076 ± 0.046	46.93 ± 7.10	19.55 ± 7.74	133.16 ± 8.61	25.55 ± 4.44	0.026 ± 0.019
Previous survey	2007	17	21.04 ± 6.13	0.336 ± 0.055	63.20 ± 3.04	61.50 ± 18.03	127.65 ± 4.74	14.29 ± 1.00	0.034 ± 0.026
Jingchun et al., 2010 [35]	2004	7^c^	30.28 ± 4.70	0.21 ± 0.03	53.91 ± 4.83	—	127.51 ± 13.92	—	—
		7^d^	28.79 ± 5.59	0.30 ± 0.01	62.81 ± 9.44	—	143.96 ± 12.37	—	—
		7^e^	36.42 ± 5.74	0.36 ± 0.08	70.09 ± 3.96	—	159.56 ± 9.88	—	—
Background^f^			5.40	0.03	16.20	—	33.90	—	—
ISQG low			65	1.5	50	80	200	20	21
ISQG high			270	10	218	370	410	70	100

Data are means ± standard deviation of sites.

ISQG, Interim Sediment Quality Guidelines. The guidelines define the ISQG-high and ISQG-low values, which represent the lower 10th percentile and 50th percentile of chemical concentrations associated with adverse biological effects, based on the results of sediment toxicity testing [37].

^a^The sampling sites in the intertidal zone.

^b^The sampling sites in the subtidal zone.

^c^The sampling sites in the mudflat.

^d^The sampling sites in the forest edge.

^e^The sampling sites in the forest.

^f^The background levels of trace metals obtained from a subsample of 41 surface sediments in a location in continental shelf area of south Taiwan Strait.

**Table 3:** Trace metals concentrations (mg/kg) in *Boleophthalmus pectinirostris* (*BP*), *Siliqua minima* (*SM*), and *Uca maracoani* (*UM*).

Biota	Cu	Zn	Pb	Cr	Cd	As	Hg
*BP* (muscle)	1.69	36.88	0.45	10.68	0.02	2.29	0.02
*BP* (liver)	7.34	105.34	0.95	12.35	0.14	2.40	0.02
*SM* (shell)	5.72	17.88	1.59	0.23	—	53.98	0.02
*SM* (muscle)	9.56	101.45	0.45	1.14	0.29	43.98	0.02
*UM*	115.99	87.50	1.50	0.72	0.62	17.65	0.01
Sediment	21.20	137.63	44.02	30.43	0.07	25.07	0.02

“—”: no detected.

**Table 4:** Contaminant factor (CF) and pollution load index (PLI) values of trace metals in sediments from Zhangjiang River Estuary mangrove zone.

Station	CF (Cu)	CF (Cd)	CF (Pb)	CF (Cr)	CF (Zn)	CF (As)	CF (Hg)	PLI
C01	0.78	0.45	2.31	0.39	1.06	3.33	0.25	0.83
C02	0.71	0.40	2.65	—	1.24	3.04	0.25	0.94
C03	0.67	0.45	3.00	0.60	1.23	3.09	0.38	0.96
C04	0.72	0.25	2.66	0.63	1.15	3.15	0.13	0.75
C05	0.71	0.20	3.14	0.74	1.18	3.20	0.50	0.94
C06	0.62	0.25	2.30	0.31	0.95	2.91	0.00	0.38
C07	0.68	0.30	2.29	0.46	1.06	3.01	0.25	0.78
C08	0.70	0.25	2.58	0.55	1.10	3.57	0.25	0.82
C09	0.67	0.25	2.47	0.48	1.14	2.81	0.13	0.70
C10	0.77	0.30	3.01	0.39	1.11	2.94	0.13	0.73
C11	0.75	0.20	2.48	0.38	1.07	3.22	0.00	0.72
C12	0.77	0.25	2.62	0.33	1.02	2.90	0.25	0.74
C13	0.54	0.20	2.91	0.32	1.11	3.44	0.00	0.85
C14	0.71	0.25	2.42	0.44	1.00	3.11	0.38	0.80
C15	0.66	0.90	2.58	0.36	1.06	3.29	1.00	1.10
C16	0.66	0.30	2.65	0.37	1.19	2.91	0.13	0.70
C17	0.84	0.20	2.53	0.49	1.18	3.31	0.13	0.72
DS01	0.68	0.45	2.89	0.39	1.15	3.40	0.25	0.86
DS02	0.83	0.55	3.74	0.20	1.11	3.31	0.88	1.02
DS03	0.62	0.30	3.46	0.28	0.94	3.71	0.25	0.77
DS04	0.34	0.55	2.55	0.42	1.08	1.98	0.13	0.66
DS05	0.52	0.40	2.30	0.23	0.98	2.50	0.13	0.62
DS06	0.57	0.15	2.55	0.26	0.98	2.85	0.50	0.70
DS07	0.67	0.95	2.75	0.14	1.10	4.00	0.13	0.75
DS08	0.58	0.15	3.51	0.37	1.05	3.09	0.63	0.81
DS09	0.62	0.35	2.84	0.18	0.96	3.64	0.25	0.72
DS10	0.56	0.20	2.54	0.18	1.11	3.26	0.25	0.65
DS11	0.62	0.20	3.03	0.18	1.05	3.52	0.38	0.72
DS12	0.67	0.30	3.03	0.46	1.06	3.56	0.13	0.75

**Table 5:** Geoaccumulation index (*I*_geo_) values of trace metals in sediments from Zhangjiangkou Mangrove National Nature Reserve.

Station	Cu	Cd	Pb	Cr	Zn	As	Hg
C01	−0.94	−1.74	0.62	−1.95	−0.49	1.15	−2.58
C02	−1.08	−1.91	0.82	0.29	−0.27	1.02	−2.58
C03	−1.16	−1.74	1.00	−1.33	−0.29	1.04	−2.00
C04	−1.05	−2.58	0.82	−1.24	−0.39	1.07	−3.58
C05	−1.07	−2.91	1.07	−1.02	−0.35	1.09	−1.58
C06	−1.29	−2.58	0.62	−2.27	−0.66	0.96	/
C07	−1.13	−2.32	0.61	−1.72	−0.51	1.00	−2.58
C08	−1.10	−2.58	0.78	−1.44	−0.45	1.25	−2.58
C09	−1.17	−2.58	0.72	−1.64	−0.40	0.90	−3.58
C10	−0.97	−2.32	1.00	−1.94	−0.43	0.97	−3.58
C11	−1.01	−2.91	0.72	−1.99	−0.48	1.10	/
C12	−0.96	−2.58	0.81	−2.20	−0.55	0.95	−2.58
C13	−1.49	−2.91	0.96	−2.22	−0.44	1.20	/
C14	−1.09	−2.58	0.69	−1.78	−0.58	1.05	−2.00
C15	−1.19	−0.74	0.78	−2.06	−0.51	1.13	−0.58
C16	−1.19	−2.32	0.82	−2.00	−0.34	0.96	−3.58
C17	−0.84	−2.91	0.75	−1.63	−0.34	1.14	−3.58
DS01	−1.14	−1.74	0.95	−1.94	−0.38	1.18	−2.58
DS02	−0.85	−1.45	1.32	−2.88	−0.43	1.14	−0.78
DS03	−1.29	−2.32	1.20	−2.44	−0.67	1.30	−2.58
DS04	−2.15	−1.45	0.77	−1.84	−0.48	0.40	−3.58
DS05	−1.53	−1.91	0.62	−2.68	−0.62	0.74	−3.58
DS06	−1.40	−3.32	0.77	−2.52	−0.61	0.93	−1.58
DS07	−1.17	−0.66	0.87	−3.46	−0.45	1.42	−3.58
DS08	−1.38	−3.32	1.23	−2.00	−0.51	1.04	−1.26
DS09	−1.27	−2.10	0.92	−3.03	−0.64	1.28	−2.58
DS10	−1.41	−2.91	0.76	−3.03	−0.43	1.12	−2.58
DS11	−1.27	−2.91	1.02	−3.05	−0.51	1.23	−2.00
DS12	−1.16	−2.32	1.01	−1.71	−0.50	1.25	−3.58

Mean	−1.20	−2.30	0.86	−2.03	−0.47	1.07	−2.49

*I*_geo_ class: 0: none, 1: none to medium, 2: moderate, 3: moderate to strong, 4: strongly polluted, 5: strong to very strong, and 6: very strong.

“/”: no data.

**Table 6:** Mean concentrations of trace metals in the sediment of the mangroves worldwide.

Location	Cr (mg/kg)	Cu (mg/kg)	Zn (mg/kg)	As (mg/kg)	Cd (mg/kg)	Hg (mg/kg)	Pb (mg/kg)	Reference
Zhangjiang River Estuary, China	30.43	21.20	133.16	25.07	0.07	0.02	125.65	This study
Pearl River Estuary, China	51.52	1.18	32.23	104.68	127.41	/	/	Bai et al., 2011 [38]
Yingluo Bay, China	16.9	0.16	32.8	5.12	49	/	/	Li et al., 2008 [39]
Mai Po mangrove swamp, HK	42.8	1.05	52.6	22.4	149	/	/	Liang and Wong, 2003 [40]
Vellar Estuary, India	16.28	9.15	0.98	9.44	39.28	/	/	Palpandi and Kesavan, 2012 [22]
Sungei Buloh Wetland, Singapore	7.06	0.18	12.3	16.6	51.2	/	/	Cuong and Obbard, 2006 [41]
Port Klang, Malaysia	24.89	1.46	96.02	60.19	72.2	63.2	/	Sany et al., 2013 [42]
Mangrove zone, French Guiana	0.28	/	0.13	1.15	2.51	/	0.41	Marchand et al., 2006 [28]
Sydney Estuary, Australia	42	0.59	95	31	156	8.1	/	Nath et al., 2014 [25]
Estero Salado mangrove, Ecuador	161.69	0.97	45.24	54.4	390.19	4.37	/	Fernández-Cadena et al., 2014 [24]

“/”: no data.

**Table 7:** Pearson correlation coefficient matrix between the trace metals and major elements and fine particles in surface sediments of the ZMNNR (*n* = 29).

	Cu	Cd	Pb	Cr	Zn	As	Hg	Clay	Silt
Cd	−0.055								
Pb	**0.620**	0.313							
Cr	0.203	−0.060	0.040						
Zn	**0.339**	0.085	**0.396**	**0.565**					
As	**0.410**	0.149	**0.402**	−0.146	−0.001				
Hg	0.155	**0.327**	0.223	−0.042	−0.022	0.098			
Clay	0.313	−0.078	0.150	0.221	**0.463**	−0.199	0.219		
Silt	0.203	**−0.363**	−0.064	**0.317**	**0.362**	**−0.424**	0.046	**0.882**	
Sand	−0.273	0.178	−0.064	−0.289	**−0.457**	0.314	−0.082	**−0.959**	**−0.957**

The bold numbers represent the significant coefficients at the level of *p* < 0.05.

**Table 8:** Total variance explained and component matrices for the trace metals in surface sediments from the ZMNNR.

Component	Initial eigenvalues			Extraction sums of squared loading			Rotation sums of squared loading		
	Total	% of variance	Cumulative%	Total	% of variance	Cumulative%	Total	% of variance	Cumulative%
1	2.33	33.27	33.27	2.33	33.27	33.27	1.98	28.27	28.27
2	1.60	22.89	56.15	1.60	22.89	56.15	1.70	24.23	52.50
3	1.15	16.45	72.61	1.15	16.45	72.61	1.41	20.11	72.61
4	0.76	10.80	83.40						
5	0.56	7.97	91.37						
6	0.38	5.38	96.75						
7	0.23	3.26	100.00						

Elements	Component matrix						Rotated component matrix^a^		
	F1	F2	F3				F1	F2	F3

Cu	0.79	0.06	−0.38				0.84	0.28	−0.04
Cd	0.36	−0.41	0.65				0.06	0.00	0.85
Pb	0.86	−0.15	−0.07				0.78	0.22	0.33
Cr	0.29	0.80	0.24				−0.07	0.87	−0.08
Zn	0.57	0.65	0.19				0.22	0.86	0.06
As	0.55	−0.43	−0.44				0.78	−0.27	0.05
Hg	0.33	−0.41	0.55				0.09	−0.03	0.75

Extraction method: principal component analysis.

Rotation method: varimax with Kaiser normalization.

^a^Rotation converged in 4 iterations.

## References

[1] Bayen S. (2012). Occurrence, bioavailability and toxic effects of trace metals and organic contaminants in mangrove ecosystems: a review. *Environment International*.

[2] Wang S.-L., Xu X.-R., Sun Y.-X., Liu J.-L., Li H.-B. (2013). Heavy metal pollution in coastal areas of South China: a review. *Marine Pollution Bulletin*.

[3] Alongi D. M. (2002). Present state and future of the world's mangrove forests. *Environmental Conservation*.

[4] Kong Q., Wang Z.-B., Shu L., Miao M.-S. (2015). Characterization of the extracellular polymeric substances and microbial community of aerobic granulation sludge exposed to cefalexin. *International Biodeterioration and Biodegradation*.

[5] Ren Z., Zhang X., Wang X. (2015). AChE inhibition: one dominant factor for swimming behavior changes of Daphnia magna under DDVP exposure. *Chemosphere*.

[6] Cai M., Wang Y., Qiu C. Heavy metals in surface sediments from mangrove zone in Zhangjiang River estuary, South China.

[7] Harbison P. (1986). Mangrove muds—a sink and a source for trace metals. *Marine Pollution Bulletin*.

[8] Mounier S., Lacerda L. D., Marins R. V., Bemaim J. (2001). Copper and mercury complexing capacity of organic matter from a mangrove mud flat environment, Sepetiba Bay, Brazil. *Bulletin of Environmental Contamination and Toxicology*.

[9] Yu R., Yuan X., Zhao Y., Hu G., Tu X. (2008). Heavy metal pollution in intertidal sediments from Quanzhou Bay, China. *Journal of Environmental Sciences*.

[10] Alvarez M. B., Domini C. E., Garrido M., Lista A. G., Fernández-Band B. S. (2011). Single-step chemical extraction procedures and chemometrics for assessment of heavy metal behaviour in sediment samples from the Bahía Blanca estuary, Argentina. *Journal of Soils and Sediments*.

[11] Caille N., Tiffreau C., Leyval C., Morel J. L. (2003). Solubility of metals in an anoxic sediment during prolonged aeration. *Science of the Total Environment*.

[12] Hill N. A., Simpson S. L., Johnston E. L. (2013). Beyond the bed: effects of metal contamination on recruitment to bedded sediments and overlying substrata. *Environmental Pollution*.

[13] Bhattacharya B. D., Nayak D. C., Sarkar S. K., Biswas S. N., Rakshit D., Ahmed M. K. (2015). Distribution of dissolved trace metals in coastal regions of Indian Sundarban mangrove wetland: a multivariate approach. *Journal of Cleaner Production*.

[14] Zenobi M. C., Rueda E. H. (2006). Adsorption of Me(II), HEDP, and Me(II)-HEDP onto boehmite at nonstoichiometric Me(II)-HEDP concentrations. *Environmental Science and Technology*.

[15] Arruda M. A. Z., Azevedo R. A. (2009). Metallomics and chemical speciation: towards a better understanding of metal-induced stress in plants. *Annals of Applied Biology*.

[16] Stewart A. R. (1999). Accumulation of Cd by a freshwater mussel (*Pyganodon grandis*) is reduced in the presence of Cu, Zn, Pb, and Ni. *Canadian Journal of Fisheries and Aquatic Sciences*.

[17] Wang L., Ren Z., Kim H., Xia C., Fu R., Chon T.-S. (2015). Characterizing response behavior of medaka (*Oryzias latipes*) under chemical stress based on self-organizing map and filtering by integration. *Ecological Informatics*.

[18] Yin L., Yang H., Si G. (2016). Persistence parameter: a reliable measurement for behavioral responses of medaka (oryzias latipes) to environmental stress. *Environmental Modeling & Assessment*.

[19] Mwevura H., Othman O. C., Mhehe G. L. (2002). Organochlorine pesticide residues in sediments and biota from the coastal area of Dar es Salaam city, Tanzania. *Marine Pollution Bulletin*.

[20] Cantillo A. Y., Lauenstein G. G., O'Connor T. P. (1997). Mollusc and sediment contaminant levels and trends in south Florida coastal waters. *Marine Pollution Bulletin*.

[21] Nordhaus I., Wolff M., Diele K. (2006). Litter processing and population food intake of the mangrove crab *Ucides cordatus* in a high intertidal forest in northern Brazil. *Estuarine, Coastal and Shelf Science*.

[22] Palpandi C., Kesavan K. (2012). Heavy metal monitoring using *Nerita crepidularia*-mangrove mollusc from the Vellar estuary, Southeast coast of India. *Asian Pacific Journal of Tropical Biomedicine*.

[23] Bodin N., N'Gom-Kâ R., Kâ S. (2013). Assessment of trace metal contamination in mangrove ecosystems from Senegal, West Africa. *Chemosphere*.

[24] Fernández-Cadena J. C., Andrade S., Silva-Coello C. L., De la Iglesia R. (2014). Heavy metal concentration in mangrove surface sediments from the north-west coast of South America. *Marine Pollution Bulletin*.

[25] Nath B., Chaudhuri P., Birch G. (2014). Assessment of biotic response to heavy metal contamination in *Avicennia marina* mangrove ecosystems in Sydney Estuary, Australia. *Ecotoxicology and Environmental Safety*.

[26] Sandilyan S., Kathiresan K. (2014). Decline of mangroves—a threat of heavy metal poisoning in Asia. *Ocean and Coastal Management*.

[27] Defew L. H., Mair J. M., Guzman H. M. (2005). An assessment of metal contamination in mangrove sediments and leaves from Punta Mala Bay, Pacific Panama. *Marine Pollution Bulletin*.

[28] Marchand C., Lallier-Vergès E., Baltzer F., Albéric P., Cossa D., Baillif P. (2006). Heavy metals distribution in mangrove sediments along the mobile coastline of French Guiana. *Marine Chemistry*.

[29] Janaki-Raman D., Jonathan M. P., Srinivasalu S., Armstrong-Altrin J. S., Mohan S. P., Ram-Mohan V. (2007). Trace metal enrichments in core sediments in Muthupet mangroves, SE coast of India: application of acid leachable technique. *Environmental Pollution*.

[30] Tam N. F. Y., Wong T. W. Y., Wong Y. S. (2005). A case study on fuel oil contamination in a mangrove swamp in Hong Kong. *Marine Pollution Bulletin*.

[31] Ma H., Song Q., Wang X. (2009). Accumulation of petroleum hydrocarbons and heavy metals in clams (*Ruditapes philippinarum*) in Jiaozhou Bay, China. *Chinese Journal of Oceanology and Limnology*.

[32] Qiu Y.-W., Yu K.-F., Zhang G., Wang W.-X. (2011). Accumulation and partitioning of seven trace metals in mangroves and sediment cores from three estuarine wetlands of Hainan Island, China. *Journal of Hazardous Materials*.

[33] Xie C. X., Ding Z. H., Gao W. Q. (2006). Distribution and speciation of Cu,Zn and Cr in mangrove sediments from zhangjiang estuary. *Journal of Xiamen University*.

[34] Zhang C. X., Sun X. L., Chen C. L. (2006). Distribution features and evaluation on potential ecological risk of heavy metals in submarine surface sediments of Zhanjiang Bay. *Journal of Zhanjiang Ocean University*.

[35] Jingchun L., Chongling Y., Spencer K. L., Ruifeng Z., Haoliang L. (2010). The distribution of acid-volatile sulfide and simultaneously extracted metals in sediments from a mangrove forest and adjacent mudflat in Zhangjiang Estuary, China. *Marine Pollution Bulletin*.

[36] Wang W., Dong C., Dong W. (2015). The design and implementation of risk assessment model for hazard installations based on AHP-FCE method: a case study of Nansi Lake Basin. *Ecological Informatics*.

[37] ANZECC/ARMCANZ (2000). *Sediment Quality Guidelines*.

[38] Bai J., Xiao R., Cui B. (2011). Assessment of heavy metal pollution in wetland soils from the young and old reclaimed regions in the Pearl River Estuary, South China. *Environmental Pollution*.

[39] Li Z., Zhang Z., Li J. (2008). Pollen distribution in surface sediments of a mangrove system, Yingluo Bay, Guangxi, China. *Review of Palaeobotany and Palynology*.

[40] Liang Y., Wong M. H. (2003). Spatial and temporal organic and heavy metal pollution at Mai Po Marshes Nature Reserve, Hong Kong. *Chemosphere*.

[41] Cuong D. T., Obbard J. P. (2006). Metal speciation in coastal marine sediments from Singapore using a modified BCR-sequential extraction procedure. *Applied Geochemistry*.

[42] Sany S. B. T., Salleh A., Rezayi M., Saadati N., Narimany L., Tehrani G. M. (2013). Distribution and contamination of heavy metal in the coastal sediments of Port Klang, Selangor, Malaysia. *Water, Air, & Soil Pollution*.

[43] Shi R., Lin J., Ye Y., Ma Y., Cai M. (2015). The level and bioaccumulation of Cd, Cu, Cr and Zn in benthopelagic species from the Bering Sea. *Acta Oceanologica Sinica*.

[44] Suresh G., Ramasamy V., Meenakshisundaram V., Venkatachalapathy R., Ponnusamy V. (2011). Influence of mineralogical and heavy metal composition on natural radionuclide concentrations in the river sediments. *Applied Radiation and Isotopes*.

[45] Hakanson L. (1980). An ecological risk index for aquatic pollution control.a sedimentological approach. *Water Research*.

[46] Maanan M., Saddik M., Maanan M., Chaibi M., Assobhei O., Zourarah B. (2015). Environmental and ecological risk assessment of heavy metals in sediments of Nador lagoon, Morocco. *Ecological Indicators*.

[47] Müller G. (1969). Index of geoaccumulation in sediments of the Rhine River. *Geojournal*.

[48] Loska K., Wiechuła D., Korus I. (2004). Metal contamination of farming soils affected by industry. *Environment International*.

[49] Szefer P., Ali A. A., Ba-Haroon A. A., Rajeh A. A., Geldon J., Nabrzyski M. (1999). Distribution and relationships of selected trace metals in molluscs and associated sediments from the Gulf of Aden, Yemen. *Environmental Pollution*.

[50] Usero J., Morillo J., Gracia I. (2005). Heavy metal concentrations in molluscs from the Atlantic coast of southern Spain. *Chemosphere*.

[51] Chen T.-B., Zheng Y.-M., Lei M. (2005). Assessment of heavy metal pollution in surface soils of urban parks in Beijing, China. *Chemosphere*.

[52] Sun Y., Zhou Q., Xie X., Liu R. (2010). Spatial, sources and risk assessment of heavy metal contamination of urban soils in typical regions of Shenyang, China. *Journal of Hazardous Materials*.

[53] Yi Y., Yang Z., Zhang S. (2011). Ecological risk assessment of heavy metals in sediment and human health risk assessment of heavy metals in fishes in the middle and lower reaches of the Yangtze River basin. *Environmental Pollution*.

[54] Clark M. W., McConchie D., Lewis D. W., Saenger P. (1998). Redox stratification and heavy metal partitioning in *Avicennia*-dominated mangrove sediments: a geochemical model. *Chemical Geology*.

[55] Tam N. F. Y., Wong Y. S. (1996). Retention and distribution of heavy metals in mangrove soils receiving wastewater. *Environmental Pollution*.

[56] Fu J., Zhao C., Luo Y. (2014). Heavy metals in surface sediments of the Jialu River, China: their relations to environmental factors. *Journal of Hazardous Materials*.

[57] McLaughlin M. J., Tiller K. G., Naidu R., Stevens D. P. (1996). Review: the behaviour and environmental impact of contaminants in fertilizers. *Australian Journal of Soil Research*.

[58] Kimura Y., Hsu H., Toyama H., Senda M., Alpert N. M. (1999). Improved signal-to-noise ratio in parametric images by cluster analysis. *NeuroImage*.

[59] Bhuiyan M. A. H., Islam M. A., Dampare S. B., Parvez L., Suzuki S. (2010). Evaluation of hazardous metal pollution in irrigation and drinking water systems in the vicinity of a coal mine area of northwestern Bangladesh. *Journal of Hazardous Materials*.

[60] Hu B., Li J., Zhao J., Yang J., Bai F., Dou Y. (2013). Heavy metal in surface sediments of the Liaodong Bay, Bohai Sea: distribution, contamination, and sources. *Environmental Monitoring & Assessment*.

[61] Lin Y.-P., Teng T.-P., Chang T.-K. (2002). Multivariate analysis of soil heavy metal pollution and landscape pattern in Changhua county in Taiwan. *Landscape and Urban Planning*.

[62] Roesijadi G., Robinson W. E., Mallins D. C., Ostrander G. K. (1994). Metal regulation in aquatic animals: mechanisms of uptake, accumulation and release. *Aquatic Toxicology. Molecular, Biochemical and Cellular Perspectives*.

[63] Canli M., Furness R. W. (1995). Mercury and cadmium uptake from seawater and from food by the Norway lobster *Nephrops norvegicus*. *Environmental Toxicology and Chemistry*.

[64] Saha M., Sarkar S. K., Bhattacharya B. (2006). Interspecific variation in heavy metal body concentrations in biota of Sunderban mangrove wetland, northeast India. *Environment International*.

[65] Segovia-Zavala J. A., Delgadillo-Hinojosa F., Vidal-Talamantes R. (2003). Mytilus californianus transplanted as upwelling bioindicators to two areas off Baja California, Mexico. *Ciencias Marinas*.

[66] Okazaki Y., Takahashi K., Asahi H. (2005). Productivity changes in the Bering Sea during the late Quaternary. *Deep Sea Research Part II: Topical Studies in Oceanography*.

[67] Huang Y., Huang X., Hou L. (2014). Molecular cloning and characterization of three novel Hemocyanins from Chinese mitten crab, *Eriocheir sinensis*. *Aquaculture*.

[68] Sivaperumal P., Sankar T. V., Viswanathan Nair P. G. (2007). Heavy metal concentrations in fish, shellfish and fish products from internal markets of India vis-a-vis international standards. *Food Chemistry*.

[69] García-Lestón Julia J., Méndez J., Pásaro E., Laffon B. (2010). Genotoxic effects of lead: an updated review. *Environment International*.

[70] Islam M. S., Ahmed M. K., Habibullah-Al-Mamun M., Masunaga S. (2015). Assessment of trace metals in fish species of urban rivers in Bangladesh and health implications. *Environmental Toxicology and Pharmacology*.

[71] Besada V., Sericano J. L., Schultze F. (2014). An assessment of two decades of trace metals monitoring in wild mussels from the Northwest Atlantic and Cantabrian coastal areas of Spain, 1991–2011. *Environment International*.

[72] Monteiro C. E., Cardeira S., Cravo A., Bebianno M. J., Sánchez R. F., Relvas P. (2015). Influence of an upwelling filament on the distribution of labile fraction of dissolved Zn, Cd and Pb off Cape São Vicente, SW Iberia. *Continental Shelf Research*.

